# *NLRP3 *E311K mutation in a large family with Muckle-Wells syndrome - description of a heterogeneous phenotype and response to treatment

**DOI:** 10.1186/ar3526

**Published:** 2011-12-06

**Authors:** Jasmin B Kuemmerle-Deschner, Peter Lohse, Ina Koetter, Guenther E Dannecker, Fabian Reess, Katharina Ummenhofer, Silvia Koch, Nikolay Tzaribachev, Anja Bialkowski, Susanne M Benseler

**Affiliations:** 1 Division of Pediatric Rheumatology, Dept. of Pediatrics, University Hospital Tübingen, Hoppe-Seyler-Straße 1, 72076 Tuebingen, Germany; 2 Institut für Laboratoriumsmedizin, Prof. Blessing, Dr. Blessing und Kollegen, Bereich Molekulargenetik, Virchowstraße 10c, 78224 Singen, Germany; 3 Division of Rheumatology, Dept. of Internal Medicine, University Hospital Tübingen, Otfried-Müller-Straße 10, 72076 Tübingen, Germany; 4 Department of Pediatrics and Pediatric Rheumatology, Olgahospital, Bismarckstraße 8, 70176 Stuttgart, Stuttgart, Germany; 5 Center for Rheumatic Diseases, Dept. of Pediatric Rheumatology, Oskar-Alexander-Straße 26, 24576 Bad Bramstedt, Germany; 6 Dept. of Pediatric Rheumatology, The Hospital for Sick Children, 555 University Avenue, Toronto, Ontario M5G 1X8, Canada

## Abstract

**Introduction:**

Muckle-Wells syndrome (MWS) is an inherited autoinflammatory disease characterized by fever, rash, arthralgia, conjunctivitis, sensorineural deafness and potentially life-threatening amyloidosis. The *NLRP3/CIAS1 *E311K mutation caused a heterogeneous phenotype of MWS in a large family. This study analyzes the clinical spectrum, patterns of inflammatory parameters and reports on response to treatment.

**Methods:**

A total of 42 patients and family members were screened for the presence of the *NLRP3 *mutation. Clinical symptoms were reviewed in all family members. Classical (erythrocyte sedimentation rate (ESR, C-reactive protein (CRP)) and novel MWS inflammatory markers (serum amyloid A (SAA), cytokines, cytokine receptor levels) were determined. Patients were treated with the IL-1 inhibitors Anakinra or Canakinumab.

**Results:**

All 13 clinically affected patients were heterozygous carriers of the amino acid substitution p.Glu311Lys/E311K encoded by exon 3 of the *NLRP3 *gene, but none of the healthy family members. Disease manifestations varied widely. Except for one child, all carriers suffered from hearing loss and severe fatigue. TNF-α, IL-6, TNF-RI, and TNF-RII levels as well as SAA were elevated in three, two, one, six and ten patients, respectively. Both clinical and laboratory parameters responded quickly and sustainedly to treatment with Anakinra or Canakinumab.

**Conclusion:**

The *NLRP3 *E311K mutation is associated with a heterogeneous clinical spectrum, which may expand the view on MWS presentation. The leading symptom was hearing loss. Pericarditis, a rare but severe clinical feature of MWS, was diagnosed in three patients. One patient had a severe course, which led to renal failure secondary to amyloidosis. IL-1 inhibition leads to rapid and sustained improvement of symptoms.

## Introduction

Mutations in the *NLRP3 *gene (formerly known as *CIAS1*) have been shown to cause a spectrum of autoinflammatory diseases including familial cold autoinflammatory syndrome (FCAS), Muckle-Wells syndrome (MWS), and neonatal-onset multisystem inflammatory disease (NOMID)/chronic infantile neurologic, cutaneous, and articular syndrome (CINCA) [1]. The least severe disease in this spectrum is FCAS, which is characterized by mild features including urticaria, arthralgia, and fever after generalized exposure to cold. Neonates and young children with the most severe clinical phenotype NOMID/CINCA, in contrast, show inflammatory central nervous system involvement among many severe organ manifestations. MWS patients can present with clinical features similar to FCAS plus severe fatigue and arthritis. These patients are commonly diagnosed once they develop progressive sensorineural hearing loss. MWS patients are at high risk for systemic amyloidosis, leading to renal failure in up to 10% to 50% of patients [2,3]. The nomenclature of these autoinflammatory diseases has been revised, summarizing the disease entities under the term CAPS (cryopyrin-associated periodic syndromes) [4].

Since the first report of genetic linkage between the *CIAS1 *gene and MWS in 1999 by Cuisset [1], a total of 127 sequence variants for *NLRP3/CIAS1 *have been identified and are registered in the INFEVERS database (http://fmf.igh.cnrs.fr/infevers/) accessible via the World Wide Web [5].

*NLRP3 *mutations are missense mutations located mostly in exon 3 and involving the so-called NACHT domain [6]. It is well recognized, however, that some patients with a classical phenotype of FCAS, MWS, or NOMID/CINCA may not have mutations in *NLRP3*, suggesting the involvement of additional genes [7,8]. To complicate matters even more, patients carrying the identical amino acid substitution may present with distinctly different clinical subtypes [6]. This strongly suggests that additional genetic and/or environmental modifying factors are required to define the clinical phenotype. This challenges the concept that these conditions are single-gene disorders.

With the advent of IL-1 inhibitors, such as Anakinra, Rilonacept and Canakinumab, successful treatment of patients with CAPS has for the first time become feasible [9-11]. Rapid resolution of acute symptoms, inflammatory parameters, and also improvement of long-term disease sequelae have been reported [12-14].

The aims of this study were: 1) to characterize the clinical phenotype in a large, 42-member family including 13 individuals carrying a *NLRP3 *E311K mutation; 2) to determine classical inflammatory markers and MWS biomarkers including pro-inflammatory cytokines and their receptors in all patients; and 3) to describe the response to IL-1 inhibition in this family.

## Materials and methods

### Index case

A 12-year-old girl presented with a two-year history of recurrent fever episodes, arthralgia, arthritis, rash, conjunctivitis, and sensorineural hearing loss. Classical inflammatory markers including CRP and ESR were strongly elevated. The diagnosis of MWS was suspected based on the clinical presentation (in particular the sensorineural hearing loss) and the elevated inflammatory markers. Genetic testing revealed a heterozygous c.931G > A mutation in exon 3 of the *NLRP3 *gene on chromosome 1q44, which results in the replacement of glutamic acid (GAG) at amino acid position 311 by lysine (AAG), thereby confirming the diagnosis of MWS. Written informed consent to publish the case presentation was obtained from the index patient's parents.

#### Study design

A single center cohort study of consecutive family members of the index case was conducted between March 2004 and January 2008. All MWS patients were followed according to a standardized assessment protocol in the institutional interdisciplinary Autoinflammatory Diseases Clinic led by experienced pediatric and adult rheumatologists (JKD, IK). Written informed consent was obtained from all family members and the study was approved by the Institutional Review Board (IRB) of the Faculty of Medicine of the University of Tübingen (REB No 326/2007B01).

### Family studies

The extended family of the index patient consisted of 42 living members, covering three generations. All 42 family members were screened by standardized questioning for MWS-associated symptoms. Genetic testing for the *NLRP3 *mutation was performed in 36 family members independent of their clinical status. Only symptomatic patients with confirmed genetic mutation received audiology assessments and ophthalmology exams and MRI studies.

### Demographics and clinical data

Demographic data included sex, ethnicity, and age at diagnosis of MWS. Detailed information was collected from standardized assessments obtained for all patients at each visit. A targeted review of the family history was conducted and included consanguinity, fever, infections, eye disease (categories: conjunctivitis, uveitis, and papillary edema), hearing loss, renal failure, hypertension, musculoskeletal symptoms (categories: arthralgia, arthritis, and myalgia), and rash (characteristics: cold-induced, urticarial, and maculo-papular).

The review of systems included global measures of patient health: 1) the Patient Global Health Score, a 10-cm visual analogue scale (VAS); 2) the Patient Mood Score (VAS), and 3) the Patient Performance Global Score (VAS). Clinical symptoms including fever (pattern and duration), headache, conjunctivitis, uveitis and papillary edema, hearing loss, oral ulcers, abdominal pain, renal involvement (proteinuria, hematuria, renal failure), musculoskeletal symptoms (arthralgia, arthritis, myalgia) and skin rash (cold-induced, urticarial, maculo-papular) were recorded. A complete physical examination assessed all organ systems. Associated conditions, MWS-related comorbidities including amyloidosis, and potential complications of MWS and its treatment were sought. A Physician Global Assessment Score (VAS) was recorded at each visit. Patient follow-up assessments were documented.

### Muckle-Wells Syndrome - Disease Activity Score (MWS-DAS)

The MWS-DAS was applied as described previously [15]. This semi-quantitative score attributes one point to the presence of mild symptoms of each score item and two points to severe symptoms. The maximum score of the MWS-DAS is 20. Disease complications and sequelae including delayed puberty and amyloidosis are recorded separately. Based on consensus judgment of all participating experts, a MWS-DAS cutoff for mild disease (< 10 points) versus severe disease (≥ 10 points) was chosen.

### Laboratory data

Standardized laboratory testing was conducted at each visit and included the classical inflammatory markers and hematology tests, ESR, CRP, white blood cell count (WBC), hemoglobin (HGB), platelet count (PTL), ferritin, and fibrinogen. Serum concentrations of biomarkers and cytokines, serum amyloid A (SAA), Interleukin 1 (IL-1), Interleukin 6 (IL-6), tumor necrosis factor alpha (TNF-α), TNF receptor I (TNF-RI), and TNF receptor II (TNF-RII) were analyzed by an enzyme-linked immunosorbent assay. Renal function parameters included serum creatinine, urea, uric acid, dipstick for blood and albumin, spot urine for alpha 1-microglobulin and alpha 2-microglobulin, and 24-hour urine for creatinine clearance and proteinuria.

### Other testing in patients with confirmed MWS

Audiology Assessment: Ear, Nose, and Throat (ENT) examination and audiogram was performed at the time of diagnosis to evaluate the status of hearing and every six months thereafter as a follow-up. The audiology examination included air-conduction thresholds for pure tone frequencies at 250 to 8000 Hz, bone conduction threshold, and tympanometry.

Ophthalmologic exam: A standardized ophthalmologic evaluation was performed at diagnosis and then every three months by an ophthalmology consultant, focusing on visual changes that are sensitive in documenting optic nerve function, conjunctivitis, and uveitis.

Magnetic resonance imaging (MRI): A gadolinium-enhanced MRI was performed at diagnosis and then every 12 months as a follow-up to assess meningitis and cellular infiltrates and the inner ear.

### Treatment

Patients received Anakinra, the chimeric monoclonal IL-1 receptor antagonist, at a dose of 1 to 2 mg/kg/day in patients < 40 kg body weight and at 100 mg/dose ≥ 40 kg body (Kineret; Amgen, Cambrige, UK). The drug was self-administered by subcutaneous injection once daily. In children with persistent disease activity the Anakinra dose was stepwise escalated to a maximum of 8 mg/kg. Concurrent non-steroidal anti-inflammatory medication was added if required.

Patients were treated with Canakinumab, the fully humanized anti-IL-1 monoclonal antibody, at a dose of 150 mg s.c. for ≥ 40 kg body weight or 2 mg/kg for < 40 kg body weight. In patients who did not achieve complete remission by day 8, Canakinumab was administered at a dose of 5 mg/kg body weight intravenously. Patients were allowed to switch anti-IL-1 therapy for lack of efficacy or for patient preference. Upon discontinuation of Anakinra a disease flare had to be awaited. The maximum wait time prior to start of Canakinumab therapy was set at 14 days.

### Statistical analysis

All clinical, laboratory, and MWS-DAS data were entered into a designated ARDIS research database. Baseline demographic data were analyzed using descriptive statistics. Characteristics of the mild MWS cohort (MWS-DAS < 10) and the severe MWS cohort (MWS-DAS ≥ 10) were compared using Student's *t*-test for continuous data and the chi-square analysis of Fisher's exact test for categorical variables. All analyses were performed using SAS statistical software (version 8; SAS Institute, Cary, NC).

## Results

### Patients

**Figure 1:**
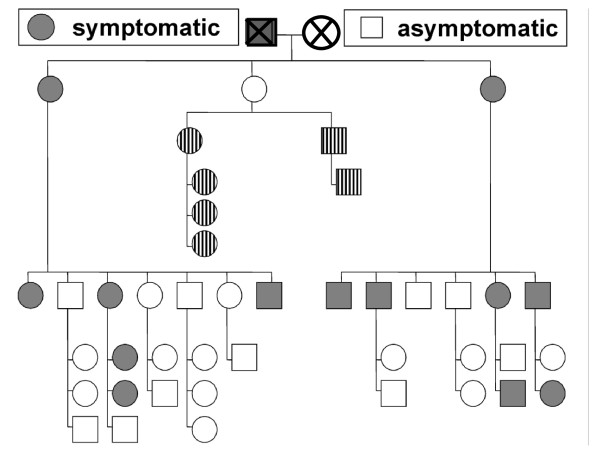
**42 family members were interviewed and examined for signs and symptoms of MWS**. Symptomatic family members are depicted in grey, asymptomatic members in white. All 13 clinically symptomatic patients are carriers of the *NLRP3 *E311K mutation (grey). Asymptomatic family members, who were not genetically tested, are marked in stripes. Clinical status of the deceased great-grandparents generation (X) was reported by children (one affected, one asymptomatic).

The study included all 42 living members of the index patient's family, 17 males and 25 females. The pedigree is depicted in Figure 1. All members completed the questionnaire for MWS-related symptoms, 36 underwent genetic testing. None of the previously described *NLRP3/CIAS1 *mutations was found. Instead, a mutation was identified in 13 of the 42 family members, causing a substitution of glutamic acid by lysine at amino acid position 311 (p.Glu311Lys or E311K). All 13 clinically affected family members were heterozygous carriers of this substitution which is encoded by exon 3 of the *NLRP3 *gene (see Table 1).

**Table 1:** Demographic characteristics and *NLRP3 *gene mutation status in an extended family with Muckle-Wells syndrome

Extended family	
Family members screened	Number = 42 (100%)
Male:female	17:25
Gender ratio	1:1.5
**Family members with MWS-associated symptoms**	
Symptomatic family members	13/42 (31%)
**Family members with confirmed *NLRP3 *mutation**	
Heterozygous carriers	13/42 (31%)
Male:female	5:8
Gender ratio	1:1.6
Age at MWS diagnosis (mean and range)	37.8 years (3.3 - 72.4)
Symptomatic family members with the E311K mutation	13/13 (100%)

MWS, Muckle-Wells syndrome

### Clinical presentation

All positively tested family members had evidence of MWS-associated symptoms. The most common one was hearing loss, seen in 12 patients (92%). Arthralgias were reported in 11 (85%), arthritis, which had improved with age, in nine patients (69%), and myalgia in 7 patients (54%). Seven patients (54%) described an erythematous, non-urticarial rash, appearing independent of cold exposure. Febrile episodes were reported only by four patients (31%), lasting on average between three and five days. The maximum temperature per episode ranged between 39°C and 40°C. All patients had constitutional symptoms, in particular chronic, severe fatigue. Seven patients (54%) reported recurrent episodes of headaches but without any corresponding abnormality in MRI examination. Ocular symptoms such as conjunctivitis, uveitis, or opticus neuritis were found in 11 patients (85%). Three patients reported a single episode of pericarditis (23%) long before the diagnosis of MWS was made. Presentation of pericarditis at that time was typical with thoracic pain and shortness of breath and treatment had consisted of NSAID and corticosteroids. Pericarditis had not re-occurred since, with or without IL-1 inhibition.

MWS-DAS was calculated at the baseline time point before the start of IL-1 inhibition for all patients. Five (38%) patients met the criteria for mild MWS activity, while severe disease was present in eight (62%). The mean MWS-DAS was 9.6 with a range between 4 and 14. Patient-derived measures at baseline had the following means and standard deviations: 1) Patient Global Health Score: 4.77 (2.20); 2) Patient Mood Score: 2.08 (0.49); and 3) Patient Performance Global: 5.85 (2.15). The Physician Global Assessment Score had a mean of 5.85 (1.52). The spectrum of clinical features is summarized in Table 2.

**Table 2:** Clinical features of all patients with the *NLRP3 *E311K mutation

	*NLRP3 *mutation, N = 13
**Clinical symptoms**	
**Constitutional symptoms**	
Fatigue	13 (100%)
Recurrent fever	4 (31%)
	
**Organ-specific symptoms**	
**Headache**	7 (54%)
**Ocular symptoms:**	11 (85%)
■ Conjunctivitis	10 (77%)
■ Uveitis	2 (15%)
■ Papillary edema	0
■ Opticus neuritis	1 (8%)
**Sensorineural hearing loss**	12 (92%)
**Oral ulcers**	6 (46%)
**Pericarditis**	3 (23%)
Abdominal pain	4 (31%)
**Renal disease/proteinuria**	10 (77%)
**Musculoskeletal symptoms:**	11 (85%)
■ Arthralgias	11 (85%)
■ Arthritis	9 (69%)
■ Myalgias	7 (54%)
**Skin symptoms:**	7 (54%)
■ Erythematous rash	7 (54%)
■ Cold-induced urticaria	0
	
**MWS Disease Activity Score (MWS-DAS)**	
Mean MWS-DAS (range)	9.6 (4-14)
mild (< 10)	5 (38%)
severe (≥ 10)	8 (62%)
	
**Global measures of health (patient-derived)**	
Patient Global Health Score VAS (mean and stdv)	4.77 (2.20)
Patient Mood Score VAS (mean and stdv)	2.08 (0.49)
Patient Performance global score Global VAS (10 = severe) (mean and stdv)	5.85 (2.15)
	
**Physician Global Assessment Score VAS** **(mean and stdv)**	5.85 (1.52)

MWS, Muckle-Wells syndrome; MWS-DAS, MWS Disease Activity Score; NLRP3, NLR family, pyrin domain containing 3; stdv, standard deviation; VAS, visual analogue scale;

### Correlation between genotype and phenotype

All 13 family members with MWS-associated clinical symptoms were found to be heterozygous carriers of the novel E311K mutation, while none of the healthy family members carried this genetic mutation. The genotype/phenotype correlation was therefore 100%. All mutation carriers reported that they had been symptomatic since childhood.

### Laboratory test results

Elevated classical inflammatory markers were found in the vast majority of patients. An increased ESR was seen in 7 patients (54%) and an elevated CRP in 13 (100%). Fibrinogen abnormalities were found in five (38%) and raised ferritin in three patients (23%). Five patients showed neutrophilia (38%).

Serum SAA levels, which may be regarded as a MWS biomarker were elevated in ten patients (77%). Increased IL-6 concentrations were observed in five patients (38%), while seven patients (54%) had high TNF-α serum levels. TNF-RI and TNF-RII were elevated in two patients. None of the patients had elevated serum IL-1 levels. Impaired renal function was rarely seen. Raised serum creatinine, urea, and uric acid were present only in the patient with renal failure. However, urine analyses demonstrated increased urine α1-microglobulin in five and increased α2-microglobulin in two patients. All laboratory test results are summarized in Table 3.

**Table 3:** Pattern of inflammatory parameters in MWS patients carrying the *NLRP3 *E311K mutation

	Number of individuals (%) with elevations and associated levels
**Classical inflammatory markers (normal)**	
**Elevated ESR **(< 22 mm/h)	
Number (%)	7/13 (54%)
Mean mm/h (stdv)	31 (20-55 mm/h)
**Elevated CRP **(< 0.5 mg/dl)	
Number (%)	13/13 (100%)
Mean (stdv)	2.25 (0.66-5.6)
**Elevated Ferritin **(< 30 μg/dl)	
Number (%)	3/13 (23%)
range	33-48 μg/dl
**Elevated Fibrinogen **(< 170 mg/dl)	
Number (%)	5/13 (38%)
range	439-540 mg/l
**Hematological abnormalities**	
Patients with hemoglobin abnormalities (%)	0/13 (0%)
Patients with leukocytosis (%)	6/13 (46%)
Mean leucocyte count/μl (range)	10,507 (7,170-21,200)
Patients with neutrophilia (%)	5/13 (38%)
Mean neutrophil count/μl (range)	7,695 (3434-18211)
Patients with thrombocytosis (%)	0/13 (0%)
**Biomarkers and cytokines**	
**Serum amyloid A (SAA) **(< 10 mg/l)	
Number (%)	10/13 (77%)
mean (range)	40.7 (10-254)
**Interleukin-1 (IL-1) **(< 5 pg/ml)	
Number(%)	0/8 (0%)
mean (range)	0.52 (0.2-1.48)
**Interleukin-6 (IL-6) **(< 5 pg/ml)	
Number (%)	5 (38%)
mean (range)	6.37 (5.9-15.7)
**Tumor necrosis factor α (TNF-α) **(< 8 pg/ml)	
Number (%)	7 (54%)
mean (range)	9.37 (8,2-16.5)
**TNF receptor I (TNF-RI) **(< 1,966 pg/ml)	
Number (%)	2 (15%)
mean (range)	1,970-> 5,000
**TNF receptor II (TNF-RII) **(< 3,170 pg/ml)	
Number (%)	2 (15%)
mean (range)	3,210-> 5,000
**Proteinuria**	
Elevated total urine protein	10/13 (77%)
Elevated alpha 1-microglobulin [μg/ml]	5/13 (28%)
range	13.2-262
Elevated alpha 2-microglobulin (mg/l)	2/13 (15%)
range	0.3-> 1.2

CRP, C-reactive protein; ESR, erythrocyte sedimentation rate; IL-1, Interleukin-1; SAA, Serum amyloid A; TNF-α, Tumor necrosis factor α; TNF-RI, TNF receptor I;

### Association with severe disease

Parameters significantly associated with severe MWS disease (MWS-DAS ≥ 10) were female gender (*P *= 0.01), musculoskeletal involvement (*P *< 0.05), eye involvement (*P *< 0.05) and the global measures Patients Global Health Score (*P *= 0.014) and Physicians Global Assessment Score (*P *= 0.0015). None of the laboratory parameters correlated significantly with disease severity.

### Treatment

Anakinra was given to 7/13 (54%) family members, 10/13 (77%) received Canakinumab. Six patients switched to Canakinumab after being treated with Anakinra. Reasons for switching from Anakinra to Canakinumab were inconvenience of daily injections and secondary treatment failure in two children. The response of clinical and laboratory parameters was rapid and profound for both substances. MWS-DAS decreased from 9.6 to 6 with Anakinra, and from 5.9 to 2.7 with Canakinumab. ESR (mm/h) decreased from 30 to 14 with Anakinra, SAA (mg/l) decreased from 35.9 to 6.8 with Anakinra and from 27.6 to 4.7 with Canakinumab and CRP (mg/dl0 decreased from 2.26 to 0.64 with Anakinra and from 2.25 to 0.23 with Canakinumab. Hearing improved in three patients, one treated with Canakinumab and two with Anakinra.

## Discussion

This study identified a mutation in the *NLRP3 *gene causing the autosomal dominantly inherited autoinflammatory Muckle-Wells syndrome. We were able to characterize the clinical phenotype, the associated laboratory parameters and the genotype in an extended family after confirming MWS in the index patient. A total of 13 family members were found to be heterozygous carriers of the E311K mutation encoded by exon 3 of the *NLRP3 *gene on chromosome 1. All 13 mutation-positive individuals were symptomatic. In contrast, none of the mutation-negative family members showed signs or symptoms associated with MWS.

This family study illustrates the heterogeneous clinical spectrum and the variability of disease severity associated with the E311K mutation. Classical inflammatory markers and MWS biomarkers were determined in all E311K-positive MWS patients. CRP was found to be strongly associated with active disease. The study also suggests that SAA is a sensitive biomarker for active MWS, although there was no linear correlation between levels of laboratory parameters and disease severity. The single amino acid substitution of glutamic acid by lysine at residue 311 leads to the synthesis of a modified cryopyrin, which in these patients may have induced the increased release of proinflammatory cytokines such as IL-6 (38%), and TNF-α (54%) as well as SAA (77%). The classical inflammatory markers CRP (100%) and ESR (54%) were also elevated.

The classical inflammatory markers including CRP were increased in all MWS patients carrying the E311K mutation, while ESR was elevated in 54%. However, the increase was only modest and depended on the actual disease activity. In comparison to E311K carriers, the median values of other cohorts [10] were higher. When elevated, the biomarkers SAA, IL-6 and TNF-α in contrast, correlated closely with the autoinflammatory state. SAA appeared to have the highest sensitivity for inflammation in this MWS family. Patients with raised SAA levels are at an increased risk of renal impairment due to progressive deposition of amyloid in the kidneys [2]. Accordingly, there is a strong correlation between SAA levels and proteinuria.

In agreement with the clinical phenotype and the moderate elevation of classical inflammatory markers, cytokine levels were only modestly raised in our cohort. Not surprisingly, serum IL-1 levels were not elevated in this cohort, reflecting the short serum IL-1 half-life as shown in previous studies [16]. Hoffman *et al*. demonstrated that serum IL-1 levels in FCAS patients with *NLRP3 *mutations were normal; however, extensive amounts of IL-1 protein and IL-1 mRNA were found in the affected skin [16].

All heterozygous carriers of the E311K mutation showed the clinical picture of MWS and had in the past sought medical attention or were even hospitalized mainly for joint problems (85%) without further specified diagnosis. All E311K mutation-positive MWS patients suffered from severe fatigue, which had significant impact on their quality of life. Of note, patients predominantly complained of arthralgias/arthritis at a younger age. In contrast, MWS-associated progressive hearing loss (92%) occurred only later in the disease course. Until the teenage years, all patients had a history of normal hearing; however, mild changes in the audiogram in the high upper frequencies were noticed as early as six years of age. As adults, all affected individuals required hearing aids. Surprisingly, fever as the leading symptom of autoinflammatory syndromes in children occurred in only 31% of our patients and then predominantly during childhood. The febrile episodes reported lasted on average between three and five days. The maximum temperature per episode ranged between 39°C and 40°C, showing the typical MWS fever pattern. There was only one MWS case with end-stage renal failure. However, mild renal impairment, as measured by urine α1-microglobulin and α2-microglobulin, was found in 77% of the patients. Three patients experienced one episode of pericarditis, which has so far only been reported in MWS-patients with the E311K mutation [17]. Following the first report [18] the *NLRP3 *E311K mutation has been described so far in 7 more patients [13,17,19] in addition to the 13 affected patients presented here. In accordance with the four patients reported by Murphy *et al*. [17], hearing loss and joint involvement was also predominant in our cohort and rash occurred only in 54% of patients. Although transient urticarial rash was described in the patient reported by Mirault [13], clinical presentation was also considered 'uncommon' by the authors with regard to the typical presentation of MWS. Pericarditis was also reported in one of four patients by Murphy *et al *[17], which may indicate that pericarditis has to be considered as one feature in MWS, at least in patients with this particular mutation (observed in 4/20 (20%) E311K patients).

Our patients responded promptly to IL-1 inhibition in contrast to the treatment response described by Murphy *et al*. Improvement of hearing loss following IL-1 inhibition was reported by three of four patients in the case series of Murphy *et al*. [17] and also in the patient described by Mirault [13]. In our cohort, 3 of the 11 treated patients showed improved hearing. The first report by Muckle and Wells described the clinical features of rash, fever, severe fatigue, arthralgia, deafness, and amyloidosis as sequelae [20]. All of these core symptoms were also found in our patients, as well as additional features such as conjunctivitis, uveitis, and headaches. Cold-induced flares, papillary edema, or frontal bossing as reported by Hawkins *et al*. [21], however, were not present in our family. In our view, the clinical presentation of patients with the *NLRP3 *E311K mutation may indeed expand the recognized spectrum of MWS features, which makes it even more difficult to give a definition of 'usual'.

There was a 100% correlation between genotype and phenotype in our MWS patients, although the phenotype was quite heterogeneous. Analysis of the genotype and phenotype performed by Aksentijevich *et al*., in contrast, demonstrated that only 2 of the 29 probands studied had the distinct clinical picture of MWS. Most of the others presented either with FCAS, FCAS/MWS, MWS/NOMID, or NOMID [22]. The clinical heterogeneity of patients carrying the same mutation is poorly understood [23]. The observation, that the identical amino acid substitution is associated with different clinical subtypes [6] suggests that *NLRP3 *mutations do not solely account for the phenotype. Reports from several groups are hypothesizing that unknown modifier genes or environmental factors can influence phenotype and disease severity [6,23,24].

Because favorable response to IL-1 inhibition in patients with the E311K mutation has been reported extensively before [11,12], only a brief description on response to treatment in this particular cohort is given in this study. In addition to the effects on clinical and laboratory parameters it is remarkable, that long-term sequelae such as hearing loss are improved by therapy in some of these patients.

## Conclusion

This is the largest cohort of MWS patients carrying the *NLRP3 *E311K mutation described to date. The heterogeneous clinical presentation of patients with this particular mutation may expand the features expected in MWS so far. Early accurate diagnosis, if possible assisted by genetic confirmation of mutation is important, since IL-1-blocking agents have been very efficacious in patients with MWS [16,21,25] and early therapeutic intervention is necessary to prevent irreversible organ damage and, in particular, hearing loss [12,13,17].

## Abbreviations

ASC: apoptosis-associated speck-like protein containing a CARD; CAPS: cryopyrin-associated periodic syndrome; CARD: caspase recruitment domain; CIAS: cold induced autoinflammatory syndrome; CINCA: chronic infantile neurologic: cutaneous: and articular syndrome; CRP: C-reactive protein; ENT: ear: nose: and throat; ESR: erythrocyte sedimentation rate; FCAS: familial cold autoinflammatory syndrome; HGB: hemoglobin; IL: interleukin; MRI: magnetic resonance imaging; MWS: Muckle-Wells syndrome; MWS-DAS: Muckle-Wells syndrome disease activity score; NACHT = NAIP: CIITA: HET-E: TP1; NF-κB: nuclear factor 'kappa-light-chain-enhancer' of activated B-cells; NLRP: nucleotide-binding oligomerization domain: leucine-rich-repeat-family: pyrin domain containing; NOMID: neonatal-onset multisystem inflammatory disease; PTL: platelet count; PYD: pyrin domain; SAA: serum-amyloid-A; TNF-α: tumor necrosis factor-α; TNF-R: tumor necrosis factor receptor; VAS: visual analogue scale; WBC: white blood cell count

## Competing interests

JKD performed clinical studies with and received honoraria from Novartis. None of the other authors declare any conflict of interest in respect to this study. There was no funding from external sources or grants received for this study.

## Authors' contributions

JKD, GD and SMB conceived of and designed the study. IK, FR, KG, NT, and AB contributed in the acquisition of data. PL carried out the molecular genetic studies. SK carried out the cytokine analysis. JKD and SMB drafted and revised the manuscript. All authors have given final approval of the version to be published.

## Acknowledgements

The authors want to thank the families for participating in the study. They would also like to acknowledge the outstanding statistical support of Pascal Tyrrell, PhD (c), University of Toronto, Division of Rheumatology, The Hospital for Sick Children, Toronto, Canada and the figure design of Peter-Michael Weber, University Childrens Hospital, Tuebingen, Germany.
